# Dobutamine stress cardiovascular MR in clinical practice: a single centre experience

**DOI:** 10.1186/1532-429X-13-S1-P227

**Published:** 2011-02-02

**Authors:** SK Khambekar, M Byrant, JS Shambrook, CP Peebles, IW Brown, SP Harden

**Affiliations:** 1 Southampton University Hospital NHS Trust, Southampton, UK

## Introduction

Dobutamine stress cardiovascular magnetic resonance (DCMR) is a robust tool for determining the presence of inducible ischaemia in patients with known or suspected coronary artery disease (CAD).

## Purpose

To assess the safety of DCMR in clinical practice in a tertiary referral centre in the UK.

## Methods

We retrospectively studied all DCMR scans performed between May 2006 and March 2009.

### CMR Protocol

CMR studies were performed using a 1.5 T (Siemens Avanto) clinical CMR scanner. After a full functional assessment using steady state free precession (SSFP) cine imaging, three short axis images together with the three long axis (2-, 3- and 4- chamber) images were selected and these were then acquired at each dose increment during a standard dobutamine-atropine protocol. Dobutamine was infused in 10 mcg/ kg/ min increments up to a maximum of 40 mcg/ kg/ min. Initial 5 mcg/ kg/ min increments were used where there was a wall motion abnormality at rest. The protocol was continued until target heart rate was achieved or until there was a recognised indication to stop prior to reaching target heart rate. Side effects and complications were recorded.

## Results

Out of 455 patients, 21(4.6%) patients were unable to undergo the procedure (claustrophobia, MR incompatible device in situ)

434 scans were performed in patients with a mean age of 64 years (range 13-86). 419 patients had a full dose study to assess for inducible ischaemia while 15 patients had a low dose protocol to determine myocardial viability.

The average dose of dobutamine required for each full dose study was 24 + 9 micrograms/ kg/ min with additional atropine used in 119 (27.4%) patients. Target heart rate was achieved in 334 (79%) patients.

Of the 85 patients who failed to achieve target heart rate, 37(43%) developed significant chest pain, requiring the infusion to be stopped. Chest pain was the commonest cardiac side effect but in only 20% of these patients was the scan stopped early. 5 patients needed in-patient overnight observation for hypotension (2), broad complex tachycardia (1) and fast AF (2). None of these had a significant rise in troponin.

Minor non-cardiac side effects occurred in 18 (4%) patients (eg nausea).

## Conclusions

Our experience suggests DCMR is a safe and feasible technique in routine clinical practice for assessing patients with suspected or known CAD and has a low complication rate.

